# Inertial Sensors for Performance Analysis in Combat Sports: A Systematic Review

**DOI:** 10.3390/sports7010028

**Published:** 2019-01-21

**Authors:** Matthew TO Worsey, Hugo G Espinosa, Jonathan B Shepherd, David V Thiel

**Affiliations:** School of Engineering and Built Environment, Griffith University, Brisbane, QLD 4111, Australia; matthew.worsey@griffithuni.edu.au (M.T.O.W.); j.shepherd@griffith.edu.au (J.B.S.); d.thiel@griffith.edu.au (D.V.T.)

**Keywords:** combat sport, technology, inertial sensor, performance

## Abstract

The integration of technology into training and competition sport settings is becoming more commonplace. Inertial sensors are one technology being used for performance monitoring. Within combat sports, there is an emerging trend to use this type of technology; however, the use and selection of this technology for combat sports has not been reviewed. To address this gap, a systematic literature review for combat sport athlete performance analysis was conducted. A total of 36 records were included for review, demonstrating that inertial measurements were predominately used for measuring strike quality. The methodology for both selecting and implementing technology appeared ad-hoc, with no guidelines for appropriately analysing the results. This review summarises a framework of best practice for selecting and implementing inertial sensor technology for evaluating combat sport performance. It is envisaged that this review will act as a guide for future research into applying technology to combat sport.

## 1. Introduction

In recent years, technological developments have resulted in the production of small, unobtrusive wearable inertial sensors. Such sensors directly measure movement both in the laboratory and in the field of play. Microelectromechanical systems (MEMS) are chip-level devices based on change in inertial movement of silicon-based arms acting as a mass and spring. From this movement, the acceleration and rotation of the device can be measured, logged and transmitted off the body [1,2]. The support requirements and the interference with normal movement are minimal. Different MEMS accelerometer designs are available; some examples include capacitance MEMS accelerometers, piezoelectric MEMS accelerometers and piezoresistive MEMS accelerometers. In combat sports, there are additional constraints as the electronics systems must survive high impact accelerations, environmental effects of temperature and humidity, wireless connectivity problems due to multiple rotations of the body, a very high sampling rate to accommodate under-sampling of high impact events, and the shape and placement of the sensors must be such that a direct hit on the sensor will not injure the participant and will not undermine the operational characteristics of the devices.

This review paper reports the current scientific literature published in the open domain to assess the current and projected use of inertial sensors in combat sports. The motivation is to provide the reader with some guidance for the possible applications of inertial sensors in their particular sport, to monitor human movement and to assist in the scoring of events (bouts).

In March 2018, a systematic literature review evaluating the use of wearable inertial sensors for sport performance was published [3]. Of the 286 included records, eight (8/286–2.8%) measured performance features in combat sport. This suggests that applying sport technology in a combat sport setting is under-researched. Combat sports are physically demanding and require high levels of skill. Therefore, performance measuring technology that can be used during training and competition could be highly beneficial. Mohamad et al. [4] conducted a systematic review with the purpose to evaluate sport science-based research surrounding Muay Thai boxing. The included records (n = 13) were divided into different research areas: sports physiology, sports biomechanics and sports psychology. The authors found that a majority of Muay Thai research investigated sport biomechanics (46.0%) followed by sport physiology (31.0%) and sport psychology (23.0%). A limitation of this review was that the type and use of technology used by the researchers was not explored. Chaabène et al. [5] reviewed literature documenting research into the physical and physiological attributes of amateur boxers. Records were included if the papers reported information regarding major fitness components of boxers, for example, body composition and somatotype, aerobic and anaerobic profiles, muscular strength and power. The review was primarily concerned with the values obtained for different performance features rather than the methodology used for measurement. A common gap in these prior reviews was to assess the technology used in combat sport research. Without a framework, ad-hoc methodologies to select and implement technology could compromise the measurement of combat sport performance with acceptable accuracy and validity. This systematic review aims to detail and to identify how inertial sensor technology has been used to monitor combat sport performance in the past and identify any gaps in existing literature worthy of investigation. This will provide researchers with a greater understanding of what work has already been done and what was achieved. In turn, there will be more clarity when designing methodologies to measure various performance features.

## 2. Materials and Methods

**Figure 1:**
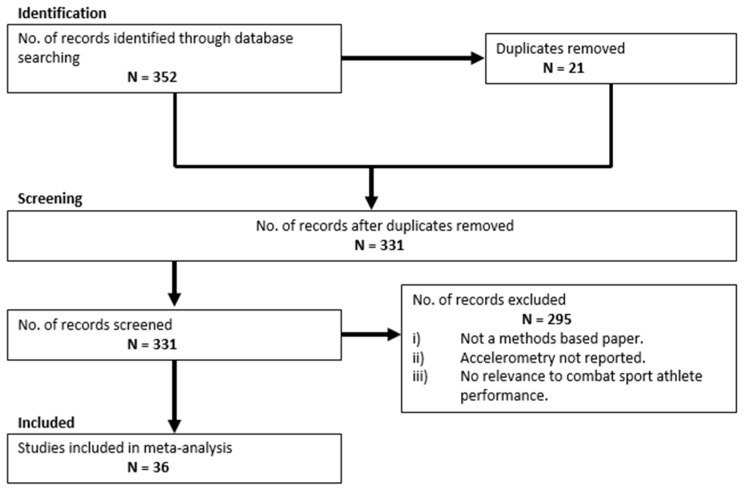
Flow diagram of study selection.

A systematic review of literature was conducted (current as of 21 September 2018) abiding to a methodology based on PRISMA (Preferred Reporting Items for Systematic Reviews and Meta-Analyses) recommendations for completing and reporting the findings of systematic reviews [6]. A total of six scholarly databases were explored (Google Scholar, Web of Science (core collection), ProQuest, Scopus, Sage Journals and Science Direct) using the keywords identified in Table 1. In order for a manuscript to be included in the final review, all of the following inclusion criteria had to be satisfied: It must be a methods-based research article from a scholarly journal (available in English), contain the use of inertial sensors and have a relevance to human performance monitoring in a combat sport setting. The initial search across all databases returned a total of 352 records, of which 295 did not meet the inclusion criteria and a further 21 were removed as duplicates. Figure 1 schematically highlights this process. 

The included papers were reviewed through extraction of information such as: (i) the geographical location of where the study was conducted; (ii) properties of the inertial sensor used in the study; (iii) the placement of the inertial sensor in the study; (iv) what algorithms were used for data processing; (v) what performance features were analysed; (vi) study design and (vii) whether other validated technologies/procedures were implemented to ensure the accuracy and validity of the investigation. 

## 3. Results

### 3.1. Journals and Years

**Figure 2:**
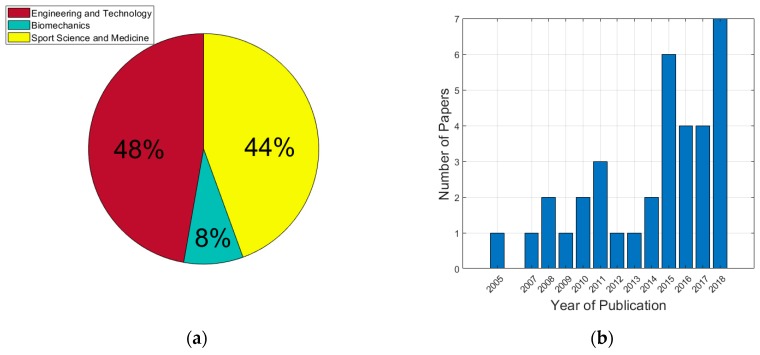
(**a**) Journal Distribution; (**b**) Publication frequency by year. The total number of papers since 2005 is 35.

The papers included in the systematic review were published in 30 different scientific journals. The journals could be divided into three fields of research: Engineering and Technology (48.0%), Sport Science and Medicine (44.0%) and Biomechanics (8.0%) (Figure 2a). The year of publication was also modelled to assess whether a trend was existent. The number of papers published each year since 2005 is shown in Figure 2b. It is clear that there has been an increase in the number of publications each year but still the published engagement in the field is relatively small at six per year.

### 3.2. Performance Features 

A wide range of factors can affect an athlete’s overall performance in combat sport. The performance factors that were examined in the captured articles were extracted (Table 1). These factors include: strike quality, strike classification, strike frequency, head impacts, automatic scoring, movement speed (footwork), power output, endurance and grappling technique. There is also significant research into the equipment used by combat sport athletes. The majority of studies aimed to enhance safety measures within the sport. It is clear that the highest research interest centered on a performance feature labelled striking quality: for example, the maximum velocity of the hand during a punch is a feature of significant interest. Another area of interest is head impacts and, indirectly, the risk of concussion of the athlete. Combat sports that are more concerned with grappling and submissions such as Jujitsu appear to be under-researched and thus these sports provide an avenue for future investigations although the methods of assessing these movements is far more complex. Inertial sensors provide time series data of three axis acceleration and three axis rotation. To extract performance features, the data must be processed mathematically through computational algorithms. The records reviewed were also compared with the techniques that were used to obtain the performance metrics (this is documented in Table 2). 

### 3.3. Data Processing Aglorithms

Signal processing of time-series data can involve many different techniques including low and high pass filtering to remove the effect of noise or drift. Frequency analysis and machine learning techniques have been applied for automatic classification of human actions. A summary of techniques used is given in Table 3.

### 3.4. Study Design and Hardware

The papers were also reviewed in respect to their study design and the properties of the inertial sensor hardware used. The study design was evaluated on the number of inertial sensor devices used, the sampling rate and operating ranges of the sensors, the positioning of the device(s), the transmission of data, the testing environment and participant selection (Table 4 and Table 5). An important additional consideration for studies testing the performance of novel hardware or innovative metrics for assessing athlete performance is a comparison with a ‘golden standard’ technology to ensure validity and reproducibility of the measurements obtained (Table 6). Commonly this standard is a multi-camera motion capture system, which can locate human position in three-dimensional space. From this data, the acceleration and rotation of the body part can be calculated and compared to the inertial sensor data. 

## 4. Discussion

### 4.1. General Trends

Recent advances in technology have led to more frequent integration of inertial sensor devices into a sporting environment. In particular, inertial sensors can be used to measure sport performance features and deliver feedback in real-time, making them ideal for athlete performance analysis both in training and competition. The three major research disciplines interested in implementing technology in a combat sport setting are Biomechanics (n = 3), Sport Science and Medicine (n = 16) and Engineering and Technology (n = 16) reported in 30 unique journals (Figure 2a). Since 2005, there has been an increase in the volume of research evaluating the use of inertial sensors in combat sport (Figure 2b). This may be due to the fact that inertial sensors are now inexpensive **[3]**, making this technology more readily available for coaching teams. The geographic spread of combat sport technology research is also diverse, with research being conducted in Australia, Poland, England, Italy, Brazil, United States of America, Germany, Serbia, Canada, New Zealand, Algeria, Slovak Republic, Republic of Korea, Portugal and Netherlands. However, the majority of the research published originated from Australia and the USA (19.0% each). The USA is home to the Ultimate Fighting Championship (UFC), the largest mixed martial arts (MMA) Promotion Company in the world [43]. MMA incorporates fighting styles from a range of disciplines; thus, it is no surprise that the USA is at the forefront of combat sport research. 

### 4.2. Performance Features

The performance feature of most interest was strike quality (16/36–44.4%). Special fitness—the ability to perform specific motor skills and functional strength level for skill-acquisition tasks, such as striking—is fundamental for combat sport athletes [13,44]. Thus, having the means to quantify and monitor the progress of an athlete’s strike quality throughout training and under fatigue would be of great interest to a coaching team. Boxing was the most commonly researched combat sport. Boxing consists of standing fist fighting, which could be another reason why a high percentage of the included records were concerned with strike effectiveness [5]. Researchers used various methods to quantify the effectiveness of a strike. All of the records (16/16–100.0%) assessed strike quality with performance measures such as strike acceleration, strike velocity and strike force [8,10,11,12,14,15,17,19,20,22,23,25,27,28,35,38]. Four of the included records (4/16–25.0%) were also concerned with striking accuracy [8,17,19,35]. A study on amateur boxing conducted by Davis et al. [45] highlighted the importance of strike accuracy. Their investigation showed that round winners do not necessarily throw more punches but instead demonstrate greater punching precision. Another four records (4/16–25.0%) investigated the time and impact duration of strikes [8,12,15,20]. A strike in combat sport can be described by the impulse-momentum change theorem. Force and time are inversely proportional and thus a combat sport athlete’s ability to cause an increase in the time duration of the collision is important. 

Ten of the included records (10/36–27.8%) investigated the use of inertial sensors to measure head impacts. Ensuring the safety of a combat sport athlete in competition is vital. Of these ten records, four (4/10–40.0%) investigated the use of instrumented head and mouth guards. Four (4/10–40.0%) of the manuscripts suggested that an application for this research would be to provide real-time feedback to bout officials and medical staff, allowing more informed clinical decisions to be made [9,13,17,37]. Three of the included records trialed the instrumented head-impact detection systems in the field: one was restricted to sparring, whereas the other two were used in a competitive setting. Two of the studies used an instrumented mouth guard, one did not register any concussions in boxing bouts [37], whereas the other record was only concerned with validation of the equipment [18]. The record restricted to sparring used an instrumented head guard and similar to Stojsih et al. [17] did not register any concussions. Two papers noted that strikes to the side of the head exhibited significantly greater peak impact accelerations compared to strikes to the front of the head [17,24]. Two of the records assessed the influence that protective equipment had on head impacts [16,24]. Schwartz et al. [24] evaluated the effectiveness of protective hand wear, foot wear and 10 ounce boxing gloves. They concluded that protective hand wear did not significantly reduce peak head accelerations; conversely, 10 ounce boxing gloves did. They reported that protective foot wear increased peak acceleration values; however, it was noted that there was a difference in the style of kicks with and without protective foot wear. McIntosh et al. [16] developed a linear impactor to test the effectiveness of head guards. The linear impactor was used to mimic punches at different velocities towards an instrumented head guard. It was found that in a velocity range of 5–9 m/s, an AIBA (International Boxing Association) approved head guard in combination with gloves offers a high level of protection to a boxer’s head. For speeds <5 m/s, the benefits of a head guard compared to just a boxing glove are small. For speeds greater than 9 m/s, a head guard offers limited protection in terms of reducing the likelihood of concussion. Fife et al. [26] assessed the head impact caused by taekwondo kicks. All of the studied kick types could be classified as concussive. The mean resultant head acceleration after a turning kick was recorded at 130 ± 51.67 g, exceeding those reported for American football and boxing. New rules in taekwondo mean that a spinning kick to the head is worth four points. Thus, medical staff must be aware that a turning kick can potentially result in concussion. All of the records concerned with concussion assessed the likelihood using the Head Injury Criterion (HIC) [13,17,26,37]. Two of the records used gyroscopes as well as accelerometers [27,30]. Gyroscopes measure angular velocity, which is an important aspect of head impact research, as the rotational movement of the head during collisions is thought to be a main contributor to concussions [13]. The records with no gyroscopes often set up an array of accelerometers or used calculations so that rotational as well as linear acceleration of the head could be measured [13,17,40]. The one record that presented concussion events was recorded for American Football athletes [37]. 

Three of the included records tested automatic scoring in combat sport, specifically boxing. Athletes scored points by landing a successful punch in a target area. This has the potential to further increase the safety of combat sport athletes. Two of the studies conducted by the same research team aimed to use instrumented head guards, vests and gloves to implement automatic scoring in box tag (a variation of boxing) [29,30]. Sensors embedded in the target areas of the equipment produce a voltage change upon impact, allowing for detection of a hit. Wrist accelerometers are also worn to detect glove impacts when changes in acceleration exceed a threshold; they are also critical for avoiding points given for self-impacts. The researchers tested their system in a competitive box tag setting and compared it to Compubox, a system used to estimate the number of punches thrown and landed in many professional boxing matches. When points for opponents were expressed as percentages, the correlation between the two scoring systems had a Pearson correlation coefficient of 0.78. Krajewskia et al. [7] also used piezoelectric sensors embedded in head guards, vests and gloves. The investigation focused on differentiating between a clean hit, blocked hit and deflected hit. The algorithm implemented scored beyond 90.0%. The accuracy of assessment can be improved with an increased sampling frequency, resulting in more samples being collected in a punch cycle. Hahn et al. [29] believe that an automated boxing scoring system should be seriously considered as a solution to existing scoring problems in amateur boxing. From the amount of research investigating head impacts in combat sports, it is evident that the safety of the athlete is of concern. The implementation of automatic scoring into combat sport can reduce the risk of injury for the athlete. Using an automatic scoring system in combination with a system that can detect the severity of head impacts, like those previously discussed, a bout can be ended before serious head injuries. For combat sports that consist of grappling as well as striking, an inertial fall detection algorithm could be manipulated to detect a successful takedown and award points accordingly.

Automatic scoring, combined with an automatic classification of the thrown strike would further justify the transition of traditional scoring to automatic. Four of the reviewed manuscripts investigated strike classification. One hundred percent of the four records used machine learning techniques. Hachaj et al. [21] used a motion capture system that consisted of 17 wearable inertial sensors to generate templates for various karate techniques. When a karate technique is performed, the captured motion is compared to the generated templates and then automatically classified. The authors state that this method worked most effectively when dealing with actions in a range of 2–4 s. The recognition rate was 94.2%, which is a good result for complex karate techniques. Two of the records used trajectory data for action recognition. Wang et al. [41] applied convolutional neural networks to Joint Trajectory Maps (3D skeleton sequences represented by 2D colour distribution images dependent on joint trajectories and their dynamics). The research did not focus on combat sport but was able to identify combat sport actions (punch right, punch left, kick right, kick left, defend) with 100.0% accuracy except for punch right which was classified correctly 96.0% of the time. Conversely, Soekarjo et al. [34] applied k nearest neighbor and linear support vector classification techniques. Both of the machine learning techniques could identify the limb used for the strike with 99.0% accuracy. However, when classifying the strike performed, this accuracy reduced to 86.0%. Accuracy was increased to 89.8%, 89.1%, 91.1% and 88.0% for right hand, left hand, right foot and left foot strikes, respectively, when using hierarchal classification. 

Ten records investigated the strength and conditioning of combat sport athletes, assessing performance features such as endurance, power output and agility. Endurance was quantified by metrics such as metabolic equivalents (METs), the intensity loads that combat sport athletes endure and fatigue-induced performance decrease. Puciato [42] used accelerometers to assess how the caloric cost and intensity of training varied between different combat sports. With the exception of Jujitsu and Capoeira, all the combat sports on average exceeded the upper value of the recommended energy expenditure to benefit health. On average, all of the combat sports reached the recommended moderate intensity of exercises to benefit health (3–6 METs), except for those training Jujitsu. Jujitsu is a combat sport focused around groundwork and, therefore, athletes involved in this sport would have to be conditioned in a different way to striking combat sports athletes.

Kirk et al. [31] conducted an investigation into measuring the workload experienced by Mixed Martial Arts (MMA) athletes using accelerometry. The researchers stated that accelerometers were capable of tracking performance and output fatigue with a high level of sensitivity. Their key finding was that MMA participants are more likely to engage in low load activities, one or two strikes, and holding within a clinch and spend less time attempting explosive movements. The main difference between winners and losers of each bout was the number of successful takedowns. 

Shepherd et al. [19] looked to assess the performance of inertial sensors for fatigue detection in boxers during intensive training. Six elite boxers were instructed to strike a wall bag as fast and hard as they could for eleven sets of five second bursts, with five seconds rest between each set. The inertial sensors were secured to the participant’s wrists using Velcro straps. The authors found that the average impact acceleration for the strikes decreased with each set (r = −0.97) and the time between same hand punches increased with each set (r = 0.89), showing an onset of fatigue. The authors also assessed the difference in pitch angle to clarify whether the fatigue caused a decrease in striking accuracy; however, the pitch angle remained constant throughout the trial. The researchers concluded that inertial sensors can detect fatigue in boxers in terms of force per punch and hand speed per punch. 

Rocha et al. [28] evaluated the endurance of taekwondo athletes using a taekwondo specific test (TSAT) and assessed the validity of the test by comparing it to the Wingate Anaerobic Test (WAnT) as a golden standard. The variables extracted from the testing were peak power output (PP), relative peak power output (RPP), mean anaerobic power (MAP), relative mean anaerobic power (RMAP), fatigue index (FI) and anaerobic capacity (AC). The TSAT also extracted the number of Bandal chagui strikes thrown in 30 s. Comparison between the two tests showed a level of agreement, especially in the variables PP, RPP, MAP, RMAP and FI. A 22.0% difference existed in AC for the two tests. The authors stated that the WAnT overestimated the anaerobic performance of taekwondo athletes, although this may be due to a decrease in motor activity due to fatigue and in turn a decrease in strike accuracy, meaning that the sensor in the center of the shield was recording lower impact values than the true force. However, the results found by Shepherd et al. [19] do not agree with this. Shepherd et al. did not observe any change in the pitch angle of the wrist-located sensor on elite level boxers. This suggests that the accuracy of two elite boxers did not decrease with fatigue.

Four records used inertial sensors to quantify the power output of combat sport athletes. Two of these records used a counter-movement jump (CMJ). A CMJ is a common measure for evaluating the explosive characteristics of elite athletes [46]. Goulart et al. [25] assessed whether there was a correlation between roundhouse kick and CMJ performance in Taekwondo. It was concluded that a strong positive significant correlation existed between CMJ height and roundhouse kick speed, although not enough to justify using a CMJ test to assess roundhouse kick performance. Rocha et al. [28] used a CMJ as part of an anaerobic fitness assessment of taekwondo athletes. The results demonstrated a significant correlation between performance in the CMJ and the number of Bandal chagui strikes thrown in 30 s (r = 0.59) and the mean anaerobic power (0.56) registered in the TSAT. This correlation can be explained by the fact that the biomechanical processes involved in a Bandal chagui strike mimics those in the CMJ (knee flexion before leg extension). 

Da Silva et al. [33] analysed the power of Brazilian Jujitsu (BJJ) athletes using an accelerometer instrumented barbell. The participants were divided into two groups, advanced and non-advanced. Initially, the athletes were tested for their 1 rep max in a bench press throw (BPT). They then performed the BPT at 30.0, 40.0, 50.0 and 60.0% of their 1 rep max. It was found that BJJ athletes produced maximum power output at 42.0% of their 1 rep max. This research is beneficial for strength and conditioning coaches of BJJ athletes. High correlations exist between maximal strength and power. High power movements, such as throws and takedowns are fundamental features of BJJ. Thus, the ability for athletes to develop significant muscular strength is important. Strength and conditioning coaches can use these findings to develop individualised strength programs for BJJ athletes, further enhancing preparation for competition. Gašić et al. [39] also used an accelerometer instrumented barbell to measure strength in athletes. The purpose of the research was to compare the explosive strength of upper extremities between athletes participating in different sports. The researchers concluded that the results did not indicate that explosive strength is specific to each group of athletes. 

One record reported the grappling technique and suggested the safest way to collide with an opponent [32]. Measurements were made using a full body inertial sensor suit. An older participant, trained in Jujitsu, was able to reduce the impact of a collision more effectively than a younger participant with significantly less training in Jujitsu. The participant trained in Jujitsu also showed repeatability in their collision signals, whereas the other participant had significantly different features in each collision. Thus, training in a combat sport, particularly Jujitsu may be beneficial, not only for combat sport athletes but the general population as it can assist in protecting the body during collisions. 

### 4.3. Algorithms

Combat sports generally involve fast, high impact movements. In order to measure these actions, specific algorithms and hardware with an appropriately high sampling rate and operating range are required. Some of the included records used commercialised measurement technology and so limited information is reported about the data processing methods. It was common for researchers (19/36–52.8%) to use data filtering when signal processing [7,10,12,13,14,16,18,19,20,21,22,23,26,31,34,36,37,41]. Of the 19 records, 13 (68.4%) reported using a low pass filter [8,9,10,13,14,16,18,20,21,22,26,34,37]. The majority of these records (10/13–76.9%) passed their signal through a low pass filter to reduce noise [8,9,10,13,14,20,21,22,26,37]. The most commonly reported type of low pass filter was Butterworth (orders 2–6) (6/13–46.2%) [10,13,26,34,37]. The cut-off frequencies for accelerometer signals ranged from 12 Hz to 1650 Hz. Only three records recorded using a low pass filter for gyroscope signals. One used a cut off frequency of 110 Hz for noise reduction [37], and one [37] used during gyroscope signal correction had a cut off frequency of 1650 Hz [18]. Another did not state the purpose of the filter but had a cut off frequency of 300 Hz [16]. Only three of the records used a high pass filter, all for noise removal [7,9,36]. 

Four of the included records performed computational algorithms on the data to obtain orientation metrics [18,19,20,21]. Hachaj et al. [21] used the Shadow 2.0 wireless motion capture system (Motion Workshop, Seattle, WA, USA). This motion capture system outputs Euler angles to describe the subject’s rotational movements. The Shadow 2.0 is a commercially available system; thus, limited information about the algorithm used to calculate the orientation data is available. The research team recalculated the Euler angles as quaternions to eliminate hindrances due to the discontinuity in rotation descriptions in the Euler angle domain. Bartsch et al. [18] used Euler angles to define the vector from the center of gravity to the point of impact on the surface head when evaluating the performance of an intelligent mouth guard. The researchers provided limited detail of how they calculated the Euler angle measurements but stated that they can be used to calculate any impact line of action—the line perpendicular to colliding surfaces. Shepherd et al. [19] used Madgwick’s attitude heading reference system (AHRS) orientation filter which uses sensor fusion to return Euler angles [47]. Minakov et al. [20] used a quaternion-based extended Kalman filter (EKF) sensor data fusion algorithm to calculate orientation. Similar to Hachaj et al. [21], the authors argue that quaternion representation is advantageous compared to Euler angles. Reasons for this included Gimbal lock-free representation, plain normalisation and computation instead of complex trigonometry. 

The use of machine learning or artificial intelligence (AI) in the sports data world has become increasingly popular. Feature labelling using video analysis is a time-consuming task. Within the papers examined, AI algorithms were embedded to predict future events based on previous information, to create real-time classifiers. Researchers who want real-time predictive analytics could use these machine learning techniques to create individualised models to both analyse and help understand performance. Three of the reviewed manuscripts used a Fourier transform to analyse the frequency components of the data. Two of these three records used a Fourier transform in conjunction with machine learning techniques to determine whether features existed in the frequency domain, to assist in developing a more accurate model. Frequency domain features observed in the included records included: signal power and using the first three coefficients of a Short Time Fourier Transform to construct a joint dynamics feature vector in fencing. Malawski et al. [36] stated that in their research, time domain features extracted from the accelerometer were the most suitable for dynamics analysis. Magnetic and gyroscope data were less relevant for sport dynamics analysis. 

### 4.4. Hardware

Table 3 shows that a diverse range of sensors were used across the 36 reviewed manuscripts. Of the included records, 20/36 (55.6%) used more than one device for measurements and 13/36 (36.1%) used a gyroscope or magnetometer as well as an accelerometer. Five out of 13 (38.5%) devices contained just an accelerometer and gyroscope and the remaining eight also incorporated a magnetometer. The highest reported accelerometer operating range was ±750 g and the lowest was ±8 g. Ten of the included records reported strike acceleration as a performance metric. The lowest recorded strike acceleration was ±8 g [22], which is different to the rest of the literature that generally reports accelerations greater than ±20 g [10,12,17,19,20,26,27,38]. The highest strike accelerations were recorded from a turning kick in Taekwondo, which ranged from 97.28 g to 162.94 g [26]. 

The sampling frequency of the measurement devices ranged from 50 Hz to 1 MHz. Minakov et al. [20] reported a 25 ms and 15 ms impact duration for a hook punch and straight punch respectively. This demonstrates that the impact duration for a strike is minute. Therefore, an appropriate sampling rate is needed for data collection. For an impact duration of 15 ms, a sampling rate of at least 66.67 Hz is required to record one sample during the impact. To analyse the biomechanical processes of a strike with high accuracy, a much greater sampling frequency is needed (perhaps greater than 1 kHz). The record that used a sampling frequency of 50 Hz [41] was interested in classification of different movements, including boxing punches. The inertial sensor was worn on the right wrist and was used in conjunction with a Microsoft Kinect camera (30 FPS). This system achieved a classification accuracy of 79.1%. If the sampling frequency was increased then the system may demonstrate an improvement in accuracy. The majority of records (26/36–72.2%) used a device with a sampling frequency >200 Hz and the modal sampling rate was 1000 Hz, suitable for combat sport analysis. 

### 4.5. Study Design 

The study design of the included records was reviewed on the placement of the sensor(s), the testing environment and the level of the participant(s) in their associated combat sport. 

#### 4.5.1. Sensor Placement

Table 5 highlights that a variety of sensor placements were used across the reviewed studies. The majority of studies (20/36–55.6%) used inertial sensor instrumented combat sport equipment. This included instrumented head guards, mouth guards, boxing gloves, vests, punching bags, contact mats, barbell and a taekwondo racket. A hindrance with instrumenting equipment such as punching bags was observed by Rocha et al. [28]. The authors speculated that the full force of a strike was not being recorded by an instrumented punching bag due to fatigue-induced decrease in motor activity. A decrease in motor activity led to a decrease in punching accuracy and thus the strike was not connecting with the target area of the sensor. This suggests an argument for instrumenting the athlete rather than the equipment when using assessing performance features dependent on strike accuracy. The remaining 16/36 (44.4%) of reviewed manuscripts instrumented the athlete. Most researchers opted for forearm/wrist/hand sensors (10/16–62.5%) when assessing strike quality. This is the most direct sensor location for upper limbs strike analysis, which in turn reduces the complexity of analysis as there is minimal force attenuation from other body structures. Additionally, boxing was the most researched combat sport, which only uses upper limb striking [5].

Four of the reviewed manuscripts used full body sensor models. Three of the studies used the commercially available MVN BIOMECH link motion capture system (Xsens, Enschede, The Netherlands). Michnik et al. [32] used the system to assess body control during professional collisions. Rocha et al. [28] were particularly focused on the hip, knee and ankle sensor arrangement and used it to assess the anaerobic fitness level of combat sport athletes. Soekarjo et al. [34] used the system to achieve automatic classification of strike techniques using limb trajectory data. One of the studies [21] used the commercially available Shadow 2.0 wireless motion capture system (Motion Workshop, Seattle, WA, USA), also to automatically classify strikes. 

Two studies utilised a device attached to the participant’s hip. Puciato et al. [42] used this position to estimate the caloric cost of different combat sport training sessions. The researchers used Caltrac Monitor accelerometers (Muscle Dynamics, Inc., Torrance, CA, USA), which have been shown to be well suited for studies of activity level of groups when bound to the hip region [48]. Saponara [22] used a hip sensor in conjunction with a wrist and ankle sensor to develop a wearable biometric performance measurement system for combat sports. Only one of the included records [31] used a sensor placed in a harness, positioned between the third and fourth thoracic vertebrae (T3 and T4). The purpose of the study was to measure the workload of mixed martial arts using accelerometry alongside time motion analysis and lactate measures. 

#### 4.5.2. Study Environment

The study environments were classified as either laboratory, training or competition setting. The most common was the laboratory setting (22/36–61.1%), followed by training (10/36–27.8%) and competition (4/36–11.1%). A controlled study is easier to implement in laboratory and training settings; however, studies conducted in competitive environments provide rare insightful data for coaching teams and athletes. 

#### 4.5.3. Participant Selection

The majority of the studies (27/36–75.0%) recruited participants with at least some experience in their associated combat sport. Of these, 11 recruited elite level athletes, that is, they had fought at a professional level or reached the highest rank (Martial Art Hierarchy) within their combat sport. The repeatability of a study increases when high-level participants are recruited as they can perform the movements with correct form. Two of the reviewed records recruited both experienced and inexperienced participants. Gašic et al. [39] assessed the difference in the explosive strength of the upper body between different sports, sport engagement and gender. Soekarjo et al. [34] evaluated whether their algorithm could automatically classify strikes and determine skill level of the strike, which it did with a 73.3% accuracy. Including inexperienced participants also increases the diversity of their algorithm, which can work for athletes and the general public. Eight of the records either did not use human participants or did not state the experience level of the participants. 

### 4.6. Future Recommendations 

It is evident that strike quality is the most commonly assessed performance feature using inertial measurements. Automatic strike classification and automatic scoring have both been investigated, although none of the reviewed records aimed to achieve both. It is believed that combining the two would be of great interest to the combat sport community. A scoring system that also registers the type and severity of the strike landed may be more justifiable than just a hit detection system. Conversely, non-striking combat sports, such as Jujitsu, are not heavily researched.

Grappling is a fundamental component of MMA. This is reinforced by the findings of Kirk et al. [31] that the number of successful takedowns was the most prominent difference between winners and losers in competitive bouts. Identifying grappling performance features within accelerometer data would be a highly complex task, suggesting why there is almost no published literature on this topic. An initial investigation using inertial sensors in conjunction with a golden standard technology, such as a retroreflective motion capture system may provide an insight into the practicality of grappling performance measurements.

The review highlighted that filtering is widely used in combat sport inertial measurement research. Most commonly, filters were used to reduce signal noise. A minority of the reviewed records used advanced sensor fusion techniques to calculate orientation values. It is believed that more research using orientation metrics such as Euler Angles or Quaternions is warranted. This will provide a deeper insight into the biomechanical processes of combat sport and athletes can use this information to improve their ability.

One of the included records used a sensor placement between the T3 and T4 [31]. This is a commonly used sensor location for performance analysis in other sports. Catapult Sports (Catapult Sports, Melbourne, Australia) offer a commercialised wearable GPS sports sensor with an integrated IMU (inertial measurement unit). The sensor is designed to output a range of performance features using the location between T3 and T4. Catapult sensors are used by 1800 teams in 35 different sports [49]. It is believed that more combat sport research should be conducted using this sensor location. This location is non obtrusive to athletes in both training and competitive settings. Research was piloted by Dunn et al. [50] to assess the safety of players wearing electronic performance tracking device (EPTS) placed near the T3 and T4 area in association football. Although the research focused on football, after a questionnaire and semi-structured interview it was highlighted that unexpected, backward falls onto the device was a common injury concern of industry experts. In combat sport, there is potential for an athlete to be knocked out or taken down. If the athlete impacts the ground sensor first, there is a chance of injury. Consequently, careful consideration should be made about the construction and dimensions of the sensor used in order to mitigate this risk. An investigation conducted by Shepherd et al. [51] demonstrated that a wrist-mounted sensor did not affect the biomechanical processes of elite netball players when shooting. Therefore, sensors placed on striking limbs for combat sport assessment may not affect the biomechanics; however, there could be risk of sensor damage during high impacts. Full body sensor models that work in a laboratory or training setting may not be suitable for competition as they could potentially hinder the athlete’s mobility. Additionally, a shift in research focus from laboratory and training to the competition-based setting is recommended. The biomechanical factors observed in competitive bouts may differ to those found in laboratory and training-based studies. Studies conducted in a competitive setting would provide more realistic data [17]. 

## 5. Operational Guidelines 

**Figure 3:**
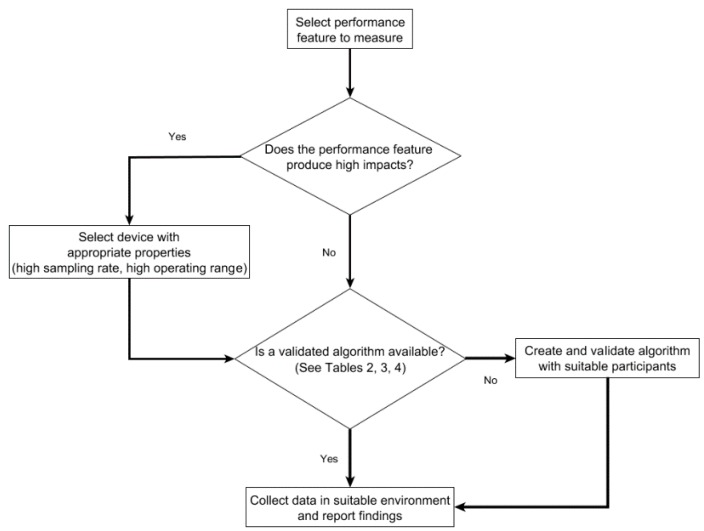
Operational guidelines methodology design in combat sport research.

There is a requirement for standardisation of data collection and analysis procedures in combat sport research. Figure 3 displays a flowchart that can assist the reader when developing methodologies for future research. Table 7 proposes the minimum hardware and data processing algorithms needed to assess different combat sport performance metrics using inertial sensors. The guidelines can help the reader in the selection of data processing algorithms, device properties, sensor positioning and validation technology.

## 6. Conclusions

This review demonstrates that inertial sensors can be used as a performance assessment tool in combat sports. It is evident that research into this field has gained momentum in the past four years. Inertial sensors were used to record performance measures associated with striking quality, automatic classification of strikes, automatic scoring, head impacts, athlete endurance, power and mobility and grappling technique. The nature of combat sport means it is essential that appropriate technology is used for investigations. The review evidenced a diverse range of hardware properties and processing algorithms that have previously shown success. From this, operational guidelines are proposed to assist readers with methodology development for future research. Future research should focus on using certain performance algorithms in combination with one another; the implementation of sensor fusion to gain more insightful measurements of combat sport biomechanics; transitioning investigations from a controlled laboratory and training environment to a competition setting, and finding a consensus on the optimal location of the sensor for these studies.

## Figures and Tables

**Table 1:** Searched databases and associated search terms used.

Database	Search Terms
Web of Science (core collection)	TS = ((Boxing OR Combat Sport) AND sports AND (Inertial Sensors OR Accelerometer OR IMU))
Scopus	TITLE-ABS-KEY ((“Boxing” OR “Combat Sport”) AND sport AND (“Inertial Sensors” OR “Accelerometer” OR “IMU”))
ProQuest	ALL (“Boxing” OR “Combat Sport”) AND ALL “sport” AND ALL (“Inertial Sensors” OR “Accelerometer” OR “IMU”)
Science Direct	(“Boxing” OR “Combat Sport”) AND “sport” AND (“Inertial Sensors” OR “Accelerometer” OR “IMU”)
Sage Journals	Anywhere (“Boxing” OR “Combat Sport”) AND anywhere (sport) AND anywhere (“Inertial Sensors” OR “Accelerometer” OR “IMU”)
Google Scholar	Boxing, OR Combat OR Sport, OR IMU, OR Inertial OR Sensor, OR Accelerometer

**Table 2:** Combat Sport Performance Features, MET (Metabolic Equivalent); TKD (Taekwondo); BJJ (Brazilian Jujitsu); MMA (Mixed Martial Arts); KB (Kickboxing).

Citation	Combat Sport	Strike Quality	Strike Classification	Strike Frequency	Head Impacts	Automatic Scoring	Movement Speed (Footwork)	Power Output	Endurance (MET, Load, Fatigue)	Grappling Technique
[7]	Boxing	x	x	x	x	✓	x	x	x	x
[8]	Boxing	✓	x	x	x	x	x	x	x	x
[9]	Boxing	x	x	x	✓	x	x	x	x	x
[10]	Boxing	✓	x	x	x	x	x	x	x	x
[11]	Boxing	x	x	x	✓	x	x	x	x	x
[12]	Boxing	✓	x	✓	x	x	x	x	x	x
[13]	Boxing	✓	x	x	✓	x	x	x	x	x
[14]	Boxing	✓	x	x	x	x	x	x	x	x
[15]	Boxing	✓	x	x	x	x	x	x	x	x
[16]	Boxing	x	x	x	✓	x	x	x	x	x
[17]	Boxing	✓	x	x	✓	x	x	x	x	x
[18]	Boxing	x	x	x	✓	x	x	x	x	x
[19]	Boxing	✓	x	✓	x	x	x	x	✓	x
[20]	Boxing	✓	x	x	x	x	x	x	x	x
[21]	Karate	x	✓	x	x	x	x	x	x	x
[22]	Karate	✓	x	x	x	x	✓	x	x	x
[23]	Karate	✓	x	x	x	x	x	x	x	x
[24]	Karate	x	x	x	✓	x	x	x	x	x
[25]	TKD	✓	x	x	x	x	x	✓	x	x
[26]	TKD	✓	x	x	✓	x	x	x	x	x
[27]	TKD	✓	x	x	x	x	x	x	x	x
[28]	TKD	✓	x	x	x	x	x	✓	✓	x
[29]	Boxtag	x	x	x	x	✓	x	x	x	x
[30]	Boxtag	x	x	x	x	✓	x	x	x	x
[31]	MMA	x	x	x	x	x	x	x	✓	x
[32]	Jujitsu	x	x	x	x	x	x	x	x	✓
[33]	BJJ	x	x	x	x	x	x	✓	x	x
[34]	KB	x	✓	x	x	x	x	x	x	x
[35]	Kung Fu	✓	x	x	x	x	x	x	x	x
[36]	Fencing	x	✓	x	x	x	x	x	x	x
[37]	Boxing/MMA	x	x	x	✓	x	x	x	x	x
[38]	Muay Thai/MMA	✓	x	x	x	x	x	x	x	x
[39]	All	x	x	x	x	x	x	✓	x	x
[40]	All	x	x	x	✓	x	x	x	x	x
[41]	All	x	✓	x	x	x	x	x	x	x
[42]	All	x	x	x	x	x	x	x	✓	x

**Table 3:** Signal processing algorithms used on the data, CT (Classification Tree); CNN (Convolution Neural Network); KNN (K-Nearest Neighbours); SVM (Support Vector Machine); DTW (Dynamic Time Warping); RNN (Radial Basis Function Neural Network).

Citation	Combat Sport	Filtering/Windowing Data	Fourier Transform (Frequency Analysis)	Machine Learning Techniques
[7]	Boxing	✓	✓	CT
[8]	Boxing	✓	x	x
[9]	Boxing	✓	x	x
[10]	Boxing	✓	x	x
[11]	Boxing	x	x	x
[12]	Boxing	✓	x	x
[13]	Boxing	✓	x	x
[14]	Boxing	✓	x	x
[15]	Boxing	x	x	x
[16]	Boxing	✓	x	x
[17]	Boxing	x	x	x
[18]	Boxing	✓	✓	x
[19]	Boxing	✓	x	x
[20]	Boxing	✓	x	x
[21]	Karate	✓	x	DTW
[22]	Karate	✓	x	x
[23]	Karate	✓	x	x
[24]	Karate	x	x	x
[25]	TKD	x	x	x
[26]	TKD	✓	x	x
[27]	TKD	x	x	x
[28]	TKD	x	x	x
[29]	Boxtag	x	x	x
[30]	Boxtag	x	x	x
[31]	MMA	x	x	x
[32]	Jujitsu	x	x	x
[33]	BJJ	x	x	x
[34]	KB	✓	x	KNN, SVM
[35]	Kung Fu	x	x	x
[36]	Fencing	✓	✓	SVM, RNN
[37]	Boxing/MMA	✓	x	SVM
[38]	Muay Thai/MMA	x	x	x
[39]	All	x	x	x
[40]	All	x	x	x
[41]	All	✓	x	CNN
[42]	All	x	x	x

**Table 4:** Properties of inertial sensor instrumentation, Number (#) of devices; OR (Operating Range); BT, (Bluetooth); RF (Radio Frequency); GPS (Global Positioning System); NS (Not Stated); x (not used).

Citation	Combat Sport	# of Devices	Accelerometer OR	Gyroscope OR	Magnetometer OR	Sampling Frequency	Transmission
[7]	Boxing	3	NS	x	x	1000 Hz	BT
[8]	Boxing	1	NS	NS	x	1000 Hz	Wired
[9]	Boxing	9	x	x	x	10 kHz	CFC 180 Filter
[10]	Boxing	1	±10 g/±100 g	x	x	1000 Hz	BT
[11]	Boxing	2	±200 g	x	x	250 Hz	BT
[12]	Boxing	2	NS	x	x	NS	Data Acquisition Card
[13]	Boxing	7	±2000 g	x	x	14.7 kHz	Data acquisition system
[14]	Boxing	2	±200 g	x	x	5000 Hz	Data Acquisition Card
[15]	Boxing	1	NS	x	x	NS	NS
[16]	Boxing	4	NS	NS	x	20k Hz	Data Acquisition System
[17]	Boxing	12	NS	x	x	1000 Hz	Wireless Transceiver
[18]	Boxing	2	±250 g	±2000 °/s	x	4000 Hz	On Board Storage
[19]	Boxing	2	±400 g	±4000 °/s	±7 Gauss	250 Hz	RF
[20]	Boxing	1	±400 g	±2000 °/s	±10 Gauss	200 Hz	BT
[21]	Karate	17	±16 g	±4000 °/s	±8 Gauss	100 Hz	Real Time
[22]	Karate	4	±200 g	x	x	1000 Hz	Real Time
[23]	Karate	1	±8 g	x	x	400 Hz	Real Time
[24]	Karate	2	±750 g	x	x	12.8 kHz	NS
[25]	TKD	1	NS	x	x	NS	NS
[26]	TKD	1	±500 g	x	x	2–4000 Hz	Wired
[27]	TKD	1	±50 g/±250 g/±500 g	x	x	8–512 Hz	USB
[28]	TKD	17	±16 g	±2000 °/s	±1.9 Gauss	240 Hz wired/60 Hz	Real Time
[29]	Boxtag	2	NS	x	x	NS	Wireless
[30]	Boxtag	2	NS	x	x	NS	Wireless
[31]	MMA	1	NS	NS	NS	100 Hz	GPS
[32]	Jujitsu	17	±16 g	±2000 °/s	±1.9 Gauss	240 Hz wired/60 Hz wireless	Real Time
[33]	BJJ	1	NS	x	x	200–500 Hz	Real Time
[34]	KB	18	±16 g (body sensors)	±2000 °/s	NS	1000 Hz/240 Hz wired/60 Hz	Real Time
[35]	Kung Fu	6	±500 g	x	x	10 kHz	NS
[36]	Fencing	1	±8 g	±2000 °/s	±8.1 Gauss	256 Hz	BT
[37]	Boxing/MMA	1	±200 g	±2000 °/s	x	1000 Hz	NS
[38]	Muay Thai/MMA	1	NS	x	x	NS	NS
[39]	All	1	NS	x	x	200–500Hz	Real Time
[40]	All	7	±70 g/±16 g/±5 g/±3 g	x	x	1 MHz	USB Data Logger
[41]	All	1	±8 g	±1000 °/s	x	50 Hz	NS
[42]	All	1	NS	x	x	NS	NS

**Table 5:** Sensor Placement, PB (Punching Bag); HG (Head Guard); MG (Mouth Guard); HM (Head Model); CM (Contact Mat); BB (Barbell); TR (Taekwondo Racket); PH (Protective Hand Wear); V (Vest); Strike Shield (SS).

Citation	Combat Sport	Full Body Model	Forearm/Wrist/Hand	Leg/Ankle	Back (Upper)	Hip	Instrumented Equipment
[7]	Boxing	x	x	x	X	X	HG,V,PH
[8]	Boxing	x	x	x	X	X	PB
[9]	Boxing	X	x	x	x	X	HG
[10]	Boxing	x	x	x	X	X	PH
[11]	Boxing	x	✓	x	x	x	x
[12]	Boxing	x	✓	x	x	x	x
[13]	Boxing	x	✓	x	x	x	x
[14]	Boxing	x	✓	x	x	x	x
[15]	Boxing	x	x	x	x	x	PB
[16]	Boxing	x	x	x	x	x	HG
[17]	Boxing	x	x	x	x	x	HG
[18]	Boxing	x	x	x	x	x	MG
[19]	Boxing	x	✓	x	x	x	x
[20]	Boxing	x	✓	x	x	x	x
[21]	Karate	✓	x	x	x	x	x
[22]	Karate	x	✓	✓	x	✓	x
[23]	Karate	x	✓	x	x	x	x
[24]	Karate	x	x	x	x	x	HM
[25]	TKD	x	x	x	x	x	TR
[26]	TKD	x	x	x	x	x	HG
[27]	TKD	x	x	x	x	x	PB
[28]	TKD	✓	x	x	x	x	SS
[29]	Boxtag	x	x	x	x	x	HG, V, PH
[30]	Boxtag	x	x	x	x	x	HG, V, PH
[31]	MMA	x	x	x	✓	x	x
[32]	Jujitsu	✓	x	x	x	x	x
[33]	BJJ	x	x	x	x	x	BB
[34]	KB	✓	x	x	x	x	x
[35]	Kung Fu	x	✓	x	x	x	x
[36]	Fencing	x	x	x	x	x	x
[37]	Boxing/MMA	x	x	x	x	x	MG
[38]	Muay Thai/MMA	x	x	x	x	x	PB
[39]	All	x	x	x	x	x	BB
[40]	All	x	x	x	x	x	HM
[41]	All	x	✓	x	x	x	x
[42]	All	x	x	x	x	✓	x

**Table 6:** Table of inertial sensor validation methods used in the included records.

Citation	Combat Sport	Validation Technology
[7]	Boxing	Video Camera
[9]	Boxing	Hybrid III Dummy
[12]	Boxing	Validation in controlled laboratory test, infrared light barrier
[13]	Boxing	High Speed Video Camera with markers
[14]	Boxing	Strain Gauge
[15]	Boxing	FiTRO Agility Plate
[16]	Boxing	Kistler uni-axial force link
[17]	Boxing	Neurocognitive assessment
[26]	TKD	8 Camera Optical Motion Capture
[29]	Boxtag	Compubox Scoring System
[31]	MMA	Video Camera/Lactate Pro Analyser
[34]	KB	Optical Motion Capture
[35]	Kung Fu	High Speed Video (2500 Hz)
[37]	Boxing/MMA	MRI, Validation in controlled laboratory test

**Table 7:** Reference guide for technology selection when conducting future research.

Performance Feature	Implementation Complexity	Minimum Hardware Requirements	Minimum Algorithm Implementation
**Strike Quality:** Acceleration/Velocity/Force Impact Accuracy	Simple Advanced	Single Accelerometer, >200 g Operating Range, >1 kHz Sampling Frequency Single IMU, >1 kHz Sampling Frequency	Threshold based peak detection Advanced orientation algorithm
Strike Classification	Advanced	IMU motion capture suit/IMU fused with optical motion capture	Machine Learning Technique
Strike Frequency	Simple	Single Accelerometer, >1 kHz Sampling Frequency	Threshold based peak detection
Head Impacts	Advanced	Accelerometer array/IMU, >200 g Operating Range, >1 kHz Sampling Frequency	Threshold based peak detection, HIC Calculation
Automatic Scoring	Advanced	Instrumented Equipment (Head Guard, Vest, Gloves)	Threshold based peak detection/Machine Learning Technique
Movement Speed	Advanced	IMU, 50 Hz Sampling Frequency	Step detection, Estimation of stride length
Power Output	Simple	Single Accelerometer (Barbell)	Calculate barbell displacement
Endurance	Simple	Single Accelerometer	MET estimation calculations
Grappling	Advanced	Validation with golden standard needed	Unknown
